# Public Health Role of Academic Medical Center in Community Outbreak of Hepatitis A, San Diego County, California, USA, 2016–2018

**DOI:** 10.3201/eid2607.191352

**Published:** 2020-07

**Authors:** Minji Kang, Sarah F. Horman, Randy A. Taplitz, Brian Clay, Marlene Millen, Amy Sitapati, Frank E. Myers, Eric C. McDonald, Shira R. Abeles, Danelle R. Wallace, Sarah Stous, Francesca J. Torriani

**Affiliations:** University of California, San Diego, California, USA (M. Kang, S.F. Horman, R.A. Taplitz, B. Clay, M. Millen, A. Sitapati, F.E. Myers, S.R. Abeles, F.J. Torriani);; County of San Diego Health and Human Services Agency, San Diego (E.C. McDonald, D.R. Wallace, S. Stous)

**Keywords:** Hepatitis A, outbreak-control program, computerized clinical decision support, viruses, San Diego, California, homelessness, illicit drug use, alcohol abuse, hepatitis, United States

## Abstract

During 2016–2018, San Diego County, California, USA, experienced one of the largest hepatitis A outbreaks in the United States in 2 decades. In close partnership with local healthcare systems, San Diego County Public Health led a public health response to the outbreak that focused on a 3-pronged strategy to vaccinate, sanitize, and educate. Healthcare systems administered nearly half of the vaccinations delivered in San Diego County. At University of California San Diego Health, the use of informatics tools assisted with the identification of at-risk populations and with vaccine delivery across outpatient and inpatient settings. In addition, acute care facilities helped prevent further disease transmission by delaying the discharge of patients with hepatitis A who were experiencing homelessness. We assessed the public health roles that acute care hospitals can play during a large community outbreak and the critical nature of ongoing collaboration between hospitals and public health systems in controlling such outbreaks.

Hepatitis A virus (HAV) is transmitted through the fecal–oral route either by person-to-person contact or by ingestion of contaminated food or water (*1*). With the availability of the hepatitis A vaccine in 1995 and the routine vaccination of children in high-incidence states (including California) since 1999 and nationally since 2006, the incidence of HAV infection has declined dramatically in the United States (*2*,*3*). Hepatitis A vaccine is highly effective; it has a seroconversion rate of ≈100% (*4*). Nevertheless, despite the substantial decline in HAV infection, sporadic cases and outbreaks continue to occur.

During 2016–2018, San Diego County, California, experienced one of the largest hepatitis A outbreaks in the United States in 2 decades (*5*). This outbreak was characterized by hepatitis A spread through person-to-person contact among persons experiencing unstable housing situations with or without illicit drug use (*5*). Since 2017, similar outbreaks have been reported in 25 states; some of the index cases in those outbreaks were linked to San Diego. As of November 1, 2019, a total of 27,634 cases, 16,679 hospitalizations, and 275 deaths have been recorded in the United States (*6*).

The public health response to the outbreak in San Diego focused on a 3-pronged strategy to vaccinate, sanitize, and educate (*7*). Local health systems, including University of California San Diego Health (UCSDH), closely and proactively collaborated with San Diego County Public Health (SDCPH) to participate in the outbreak control initiatives. We report the public health contribution of the academic medical center through the implementation of hospital-level prevention and outbreak management activities.

## Methods

### Study Setting

**Figure 1:**
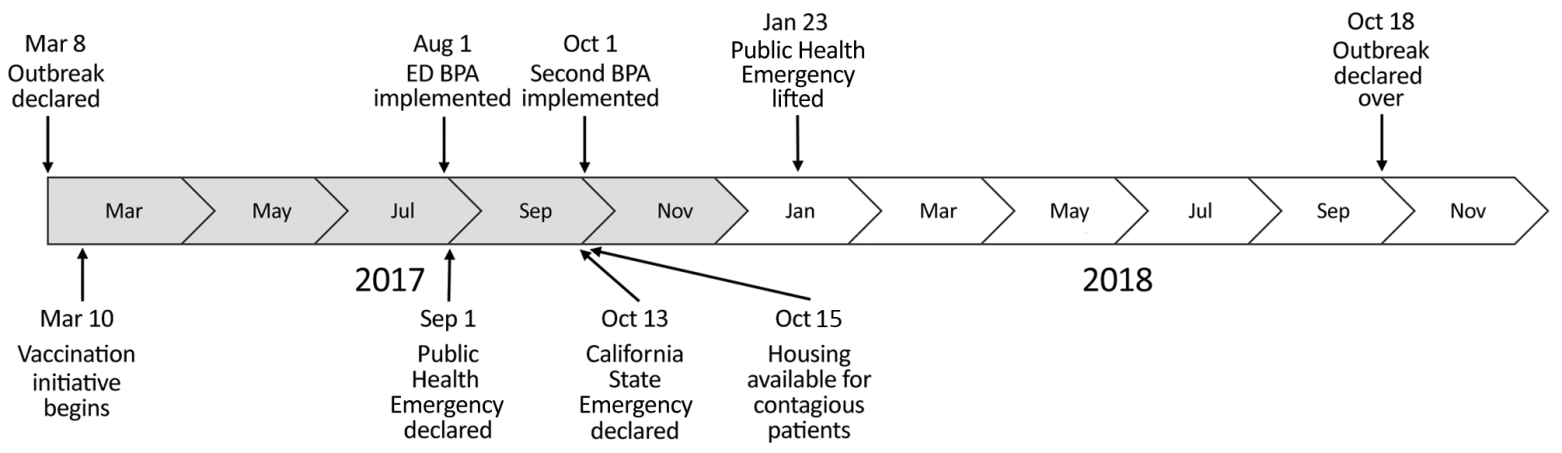
Timeline of hepatitis A outbreak, San Diego County, California, USA, 2017–2018. BPA, best practice advisory; ED, emergency department.

Our study was a retrospective review of hepatitis A diagnoses and vaccinations administered by SDCPH and UCSDH. SDCPH first declared a hepatitis A outbreak on March 8, 2017, and traced the first case to November 22, 2016 (Figure 1). The outbreak control vaccination initiatives began on March 10, 2017. SDCPH declared a public health emergency on September 1, 2017, and California health officials declared a state of emergency on October 13, 2017 (Figure 1). A declining number of cases resulted in lifting of the public health emergency on January 23, 2018. SDCPH declared the outbreak over on October 18, 2018 (*7*) (Figure 1).

UCSDH comprises 2 geographically distinct campuses within the same healthcare system. The Hillcrest campus of UCSDH is a 386-bed hospital in the urban center of the city of San Diego and is located at the epicenter of the acute hepatitis A outbreak. The Institutional Review Board at University of California San Diego reviewed this study and deemed it to be exempt from approval as a category 4 study.

### Implementation of Hospital-Level Outbreak-Control Initiatives

Hospital-level prevention and outbreak management focused on a similar strategy. The strategy comprised 3 components: vaccinate, sanitize, and educate.

#### Vaccination

Vaccination initiatives began with self-identified homeless patients seeking care at the UC San Diego Medical Center Hillcrest emergency department (ED) upon outbreak recognition in March 2017. To optimize vaccination administration, a best practice advisory (BPA), a customized alert in the electronic health records (EHR) (EpicSystems, https://www.epic.com), was constructed to flag the charts of self-identified homeless patients starting August 1, 2017. This BPA prompted providers to order the hepatitis A vaccine as described in Castillo et al. (*8*). From June 2017 through full implementation of the intervention in homeless persons in October 2017, these efforts were expanded with a second BPA, designed to identify patients with >1 of the following risk factors: homelessness, illicit drug use, alcohol abuse, cirrhosis, hepatitis B infection, and hepatitis C infection. This second BPA was constructed to increase preexposure vaccination efforts during inpatient hospitalizations at both campuses or when patients entered the care system in the ambulatory clinics or urgent care centers at UCSDH. Patients were not tested for HAV immunity before vaccination, but with each vaccination ordered, we reviewed the countywide vaccination registry for prior vaccinations. In addition, we contacted a subgroup of high-risk patients using the online patient portal for prioritized vaccination.

In May 2017, acute hepatitis A developed in a healthcare worker at UCSDH. The healthcare worker had provided care to several patients hospitalized with acute HAV infection and did not report any risk factors for hepatitis A other than occupational exposure. According to SDCPH, 7 additional healthcare workers acquired acute hepatitis A occupationally throughout San Diego County. Thus, healthcare personnel were deemed to be at risk, and SDCPH supported the recommendation by the UCSDH healthcare epidemiologist to offer vaccines at no charge to healthcare workers. Priority was given to healthcare providers with direct patient contact, along with environmental service workers and food handlers employed by the hospital, given the possibility of HAV transmission through ingestion of contaminated food or water. Hepatitis A vaccine and seasonal influenza vaccine were offered together as peer-to-peer vaccines during hospitalwide influenza vaccination drives, and hepatitis A vaccine was not denied to any healthcare workers requesting vaccination.

#### Sanitation

Enhanced contact precautions were implemented in patients with acute HAV infection during the outbreak period. These precautions were instituted in the setting of difficult-to-control diarrhea in patients with HAV infection, which most likely contributed to the documented transmission to healthcare workers who had direct contact with patients. Enhanced cleaning and disinfection were applied to rooms of patients with hepatitis A. Given some data suggesting that quaternary ammonium compounds provide insufficient virucidal disinfection (*9*), rooms occupied by patients with hepatitis A were cleaned daily with chlorine bleach products using the same disinfection procedures used for rooms occupied by patients with *Clostridioides difficile* infection. Potluck meals on hospital units were temporarily discontinued to further reduce the possibility that healthcare workers would acquire hepatitis A at work. In addition, because the outbreak centered on poor sanitary conditions, personal hygiene kits provided by SDCPH, which included hand sanitizers, soaps, cleansing wipes, bottled water, informational flyers, and waste bags, were widely distributed to at-risk patients in EDs and inpatient hospitalizations. Because the population at risk lacked regular access to sanitary living conditions, SDCPH coordinated with hospital staff on discharge planning. Patients who were potentially contagious and homeless remained hospitalized until they were able to be discharged to a temporary shelter with private restrooms. Starting October 2017, SDCPH contracted with a hotel for housing of infectious patients. All hotel staff who interacted with patients were vaccinated, and rooms were disinfected using a standard protocol.

### Education

Several articles and communications on hepatitis A were published and disseminated in UCSDH patient-oriented email newsletters to educate the general public. Nurses distributed information about hepatitis A, along with personal hygiene kits, to at-risk patients. Healthcare personnel were informed of the ongoing HAV outbreak and employee vaccination clinics through regularly scheduled UCSDH communications emails.

### Data Collection

We defined a hepatitis A case as illness that met the clinical case definition (an acute illness with a discrete onset of any signs or symptoms consistent with acute viral hepatitis and either jaundice or elevated serum alanine aminotransferase or aspartate aminotransferase) with laboratory criteria for diagnosis (positive IgM) or an epidemiologic link to a person with laboratory-confirmed hepatitis A (*10*). We obtained the number of hepatitis A cases in San Diego County during November 1, 2016–October 31, 2018, from SDCPH. We obtained the number of hepatitis A cases diagnosed at UCSDH from the infection prevention/clinical epidemiology unit and Epic Icon (EpicSystems), a data-mining software used in infection surveillance. We further stratified hepatitis A cases diagnosed at UCSDH on the basis of whether patients required inpatient hospitalization. We derived length of stay using Epic Icon.

We obtained the number of hepatitis A vaccines administered in San Diego County March 1, 2017–October 31, 2018, through the vaccination initiative from SDCPH. To collect the number of vaccinations administered at UCSDH, we used Epic Slicer Dicer (EpicSystems), a self-service analytics tool within the Epic EHR. We further stratified vaccinations administered at UCSDH according to the locations in which vaccinations were delivered (inpatient hospitalization, urgent care, ambulatory clinic, ED, or occupational health) and the patients’ risk factors (homelessness, illicit drug use, alcohol abuse, hepatitis B virus infection, hepatitis C virus infection, HIV infection, cirrhosis) within the EHR-based registries. The risk factors were not mutually exclusive, and we stratified data to identify patients with >1 risk factors.

## Results

### Hepatitis A Cases

**Figure 2:**
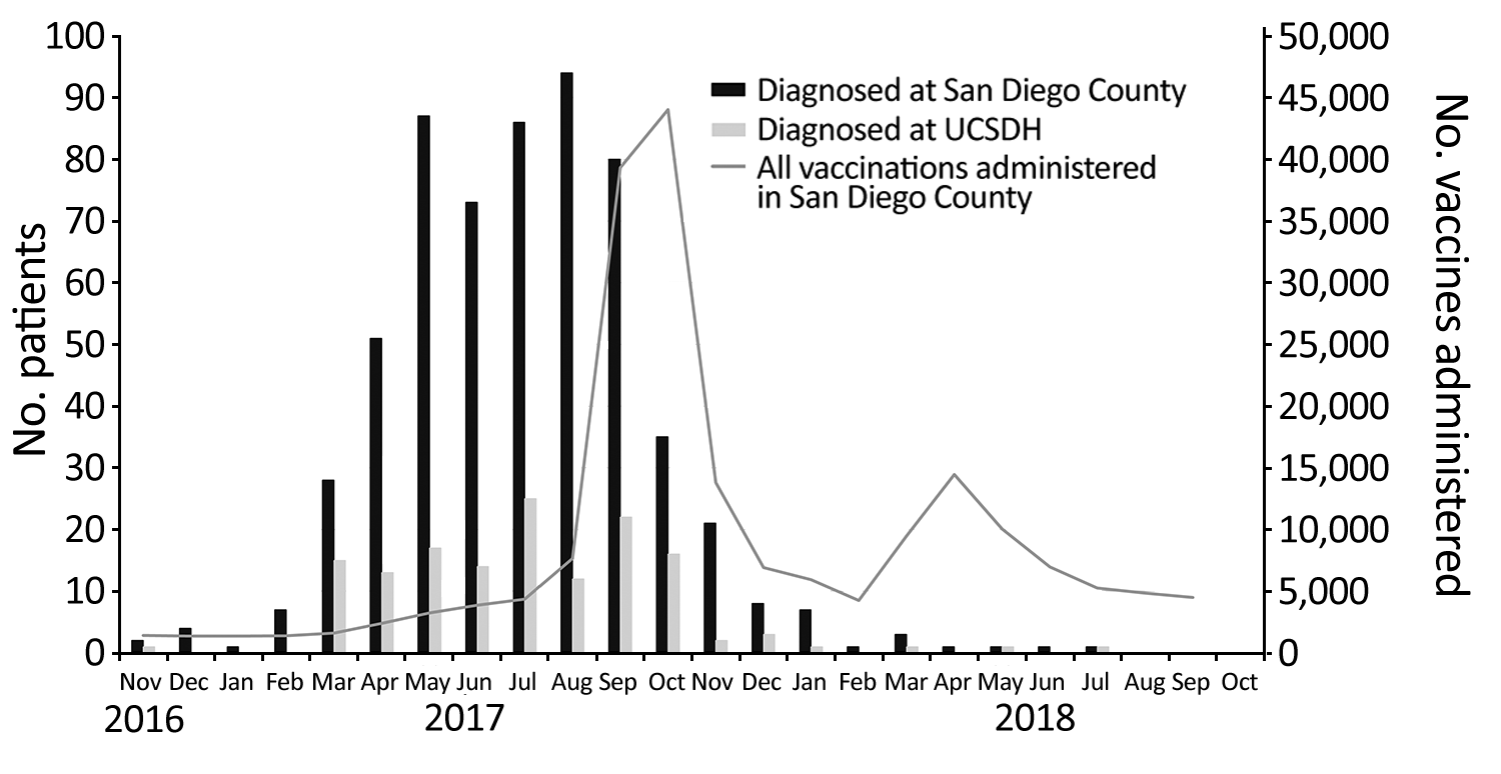
Monthly trend of hepatitis A cases in San Diego and UCSDH and all vaccinations administered in San Diego County, California, USA, 2016–2018. UCSDH, University of California San Diego Health.

**Figure 3:**
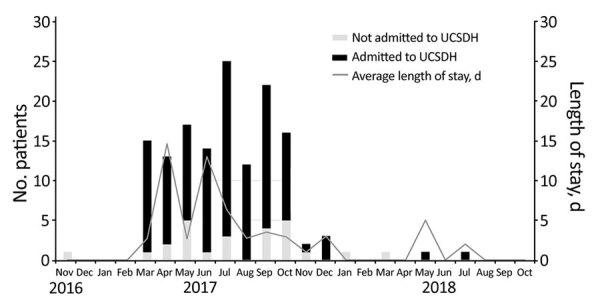
Monthly trend of persons with hepatitis A admitted to UCSDH and mean length of stay, San Diego, California, USA, 2016–2018. UCSDH, University of California San Diego Health.

During November 1, 2016–October 31, 2018, a total of 592 confirmed or probable outbreak-associated cases of HAV infection occurred in San Diego County. Although the initial cases of hepatitis A could be traced back to November 2016, the outbreak was recognized in March 2017, and cases peaked in August 2017 (Figure 2). During the 2-year period, acute hepatitis A was diagnosed in 144 patients at UCSDH. Cases began in March 2017 and peaked in July 2017 (Figures 2, 3). Among these 144 patients, 119 (83%) were hospitalized (Figure 3). In comparison, before the outbreak period (November 1, 2012–October 31, 2016), acute hepatitis A was diagnosed in 9 patients at UCSDH, of whom 5 (56%) were hospitalized.

The mean length of stay for the 119 patients admitted to UCSDH was 6.0 days (median 3.0 days, range 1–57 days) (Figure 3). Mean length of stay for all patients admitted to UC San Diego Medical Center was 5.38 days during November 1, 2016–October 31, 2018. Starting October 2017, SDCPH contracted with a hotel for housing of infectious patients. During November 1, 2016–September 31, 2017, before the availability of housing, mean length of stay for the 102 patients hospitalized was 6.4 days (range 1–57 days) (Figure 3). In comparison, during October 1, 2017–October 31, 2018, after housing became available, 17 patients were hospitalized; mean length of stay was 3.8 days (range 1–17 days) (Figure 3).

### Vaccine Administration

During March 1, 2017–October 31, 2018, a total of 207,862 hepatitis A vaccines were administered in San Diego County (Table 1). Vaccination efforts in San Diego County began in March 2017, sharply increased in August 2017, and peaked in October 2017. A second peak occurred around April 2018, when the second dose, which is typically given 6–12 months after the primary vaccination, was due (Figure 2). Among the 207,862 vaccines delivered in San Diego, San Diego County administered 57,052 (27%) vaccines: 23,620 (11%) by mass vaccination events, 5,820 (3%) by foot teams, and 848 (<1%) by mobile vans (Table 1). The remaining 150,810 (73%) vaccines were administered by noncounty providers; 99,931 (48%) were administered by healthcare systems, which included EDs, outpatient clinics, inpatient hospitals, and urgent care centers. Occupational health units throughout San Diego County administered 7,831 (4%) vaccines (Table 1).

**Table 1:** Adult hepatitis A virus vaccinations registered in the vaccination registry, San Diego, California, USA, March 1, 2017–October 31, 2018

Vaccination reason or provider	No. (%)
County	
Postexposure prophylaxis	1,026 (0.5)
Jail	9,862 (4.7)
Psychiatric hospital	522 (0.3)
Public health center	13,584 (6.5)
Public health clinic	1,770 (0.9)
Field event	
Mobile van	848 (0.4)
Foot team	5,820 (2.8)
Point of dispensing/mass vaccination	23,620 (11.4)
Noncounty	
Federally qualified health center	30,877 (14.9)
Healthcare system	99,931 (48.1)
Pharmacy	12,171 (5.9)
Occupational health entity	7,831 (3.8)
Total	207,862 (100)

**Figure 4:**
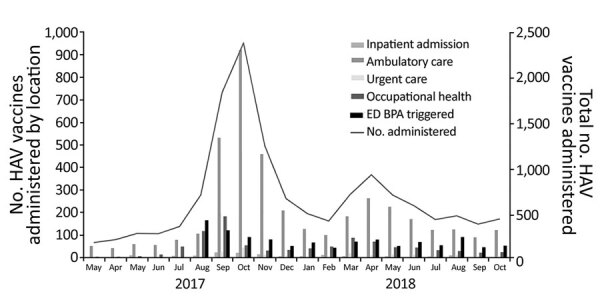
Location and monthly trend of HAV vaccinations administered at University of California San Diego Health, San Diego, California, USA, 2017–2018. BPA, best practice advisory; ED, emergency department; HAV, hepatitis A virus.

**Figure 5:**
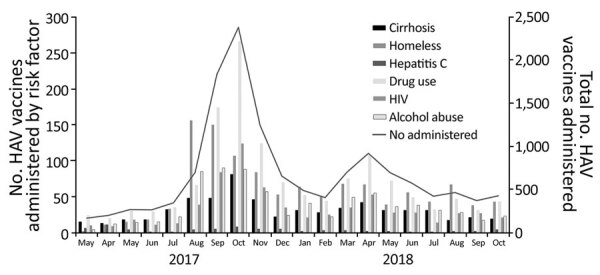
Risk factors and monthly trend of HAV vaccinations administered at University of California San Diego Health, San Diego, California, USA. HAV, hepatitis A virus.

At UCSDH, 10,324 vaccines were administered during March 1, 2017–October 31, 2018; 9,288 hepatitis A vaccine (Havrix; GlaxoSmithKline, https://www.gsksource.com) doses and 1,036 recombinant hepatitis A and B vaccine (Twinrix; GlaxoSmithKline) doses were administered (Table 2; Appendix Figure). Similar to vaccination efforts in the county, vaccinations at UCSDH increased sharply in August 2017 and peaked in October 2017 and then peaked again in April 2018 (Figures 4, 5). In comparison, during March 1, 2016–February 28, 2017, before the outbreak-control vaccination initiatives, a mean (± SD) of 118.7 (± 17.5) hepatitis A vaccines (Havrix) and 55.8 (± 11.8) recombinant hepatitis A and B vaccines (Twinrix) were administered each month. Most vaccines were delivered in ambulatory care clinics (7,700 [75%]), followed by urgent care centers (1,208 [12%]) and occupational health (961 [9%]) (Table 2; Figure 4). Illicit drug use was the most common risk factor (2,477 [24%]) of patients who were vaccinated at UCSDH, followed by homelessness (1,385 [13%]) and alcohol abuse (970 [9%]) (Table 2; Figure 5). For patients experiencing homelessness, vaccinations peaked in August 2017; for patients with risk factors of illicit drug use, alcohol abuse, HIV, and cirrhosis, vaccination peaked in October 2017 (Figure 5). Vaccination rates remained unchanged throughout the vaccination initiative for patients with chronic infections from hepatitis B, hepatitis C, or both (Figure 5).

**Table 2:** Characteristics of adults receiving hepatitis A virus vaccine administered at University of California San Diego Health, March 1, 2017–October 31, 2018*

Characteristic	No. vaccines administered		
	Hepatitis A, n = 9,288	Hepatitis A and hepatitis B, recombinant, n = 1,036	Total (%), N = 10,324
Location administered			
Inpatient admission	135	4	139 (1)
Urgent care	1,091	117	1,208 (12)
Ambulatory clinic	6,818	882	7,700 (75)
Occupational health	865	96	961 (9)
ED BPA triggered	1,369	5	1,374 (13)
Risk factors			
Homelessness	1,357	28	1,385 (13)
Illicit drug use	2,247	230	2,477 (24)
Alcohol abuse	873	97	970 (9)
Hepatitis C infection	55	49	104 (1)
Hepatitis B infection	0	0	0 (0)
HIV infection	444	141	585 (6)
Cirrhosis	411	127	538 (5)

*ED, emergency department; BPA, best practice advisory.

A total of 882 (85.1%) of 1,036 Twinrix doses were administered in ambulatory primary and specialty care clinics. Another 117 (11%) were provided in the urgent care setting. The Twinrix vaccination series was administered to established patients who also met criteria for hepatitis B vaccination, such as patients with HIV infection, hepatitis C infection, end-stage liver disease, or active illicit drug use, and who were expected to return to complete the series. A second peak of Twinrix administration occurred 6 months later in these same clinics. In contrast, Twinrix was administered twice at the UCSD Free Clinic, which focused on care of the uninsured community.

## Discussion

This study highlights the public health contribution of an acute care hospital to prevention and outbreak management activities during a hepatitis A outbreak. Unlike many other counties in California, San Diego does not have a county hospital; therefore, local acute care hospitals are actively involved in the care of vulnerable and uninsured populations. This outbreak and subsequent similar ones across the United States have been characterized by direct human-to-human spread and poor sanitary conditions disproportionately affecting the homeless population and to some extent illicit drug users (*7*,*11*–*13*). In response, the Advisory Committee on Immunization Practices recently added homelessness as an indication for hepatitis A vaccination (*14*,*15*).

Because this outbreak affected persons with limited access to routine medical care, outbreak management and prevention efforts were particularly challenging. Nevertheless, close coordination between public health and the city, behavioral health, acute care hospitals at the epicenter of the epidemic, and pharmacies helped contain the outbreak. Healthcare systems provided nearly half of the vaccinations administered in San Diego County. Although 75% of vaccinations administered at UCSDH were delivered at ambulatory clinics, and similar efforts occurred in other outpatient clinics in San Diego (*16*), proactive vaccination administration in EDs and urgent care settings or during inpatient hospitalizations with real-time access to analytics and clinical informatics support were crucial given that highest-risk population most likely had limited access to routine outpatient care (*8*,*17*).

Data are sparse on the use of acute care inpatient facilities as a setting for an aggressive outbreak-control vaccination program. However, programs to receive catch-up vaccinations among hospitalized children, as well as routine vaccinations against pneumococcus and influenza during inpatient hospitalizations, have been shown to be effective (*16*–*18*). Similarly, ED-based vaccination programs for pneumococcus and influenza have proven successful (*19*–*21*). Because EDs and urgent care may be the sole contact of a disenfranchised population with the medical system, data on EDs partnering with public health departments to administer outbreak-control vaccinations have increased (*8*,*17*,*22*).

As healthcare systems become an important site for vaccination administration, optimizing EHR support tools is crucial in increasing vaccination rates. Previous efforts focused on EDs only, but for this response, UCSDH implemented EHR-based alerts across the continuum of care to achieve higher vaccination rates (*8*). Although the direct effects of the alerts cannot be measured, vaccination of the homeless population peaked in August 2017 when the ED-focused alert was first implemented, whereas vaccination of other at-risk populations peaked in October 2017, when the second BPA alert with the wider range of risk factors was implemented across all care settings. Prior studies have shown that both computerized reminders and standing orders are effective in increasing influenza and pneumococcal vaccination rates (*23*,*24*).

In addition, although HAV infection is not typically viewed as a healthcare-associated infection, 8 unvaccinated healthcare personnel acquired hepatitis A in the healthcare setting during this outbreak. The Centers for Disease Control and Prevention does not recommend routine hepatitis A vaccination of healthcare workers because healthcare-associated HAV infection is considered infrequent. Instead, because hepatitis A is transmitted through the fecal–oral route, the Centers for Disease Control and Prevention recommends routine infection control precautions with proper hand hygiene to prevent transmission to hospital staffs (*25*). However, continued and prolonged exposure to patients with acute hepatitis A infection can result in disease in unvaccinated healthcare workers; therefore, vaccination of healthcare personnel can be considered in outbreak settings.

Finally, compared with prior outbreaks, in this outbreak, disproportionate numbers of patients with acute hepatitis A required inpatient hospitalization. Prior studies have reported that hospitalization rates for all reported hepatitis A cases were 33%–43% during 2005–2011 (*6*,*26*–*29*). Before the outbreak, during November 1, 2012–October 31, 2016, five (56%) of 9 patients in whom acute hepatitis A was diagnosed required inpatient hospitalization. During the outbreak we report, 83% of all hepatitis A patients at UCSDH were admitted, and their mean length of stay was of 6.0 days. Given that this outbreak centered on homelessness and poor sanitation, hospitalization of patients with hepatitis A most likely prevented continued human-to-human transmission through prolonged viral shedding in the homeless community. Starting in October 2017, SDCPH contracted with a hotel to arrange housing for potentially infectious patients and coordinated with hospital staff on discharge planning. Designation of protected facilities increased housing capacity and decreased the length of hospitalization for acutely infected homeless patients.

Limitations to this study include its single-center and retrospective study designs. Although other local acute care centers participated in the outbreak control initiatives, we do not have information about the number of vaccinations administered and hepatitis A cases diagnosed at other facilities. In addition, the retrospective nature of the study precludes a precise assessment of the direct effect of BPAs on the number of hepatitis A vaccines administered and the effect on vaccinations by risk factors and on the scope of the sanitation efforts within the UCSDH system. Finally, the effect of inpatient hospitalizations on limiting human-to-human transmission in the homeless community cannot be definitively demonstrated. Communitywide initiatives, in addition to hospital-level efforts, might have influenced the actions of UCSDH providers.

Although the hepatitis A outbreak in San Diego and California was ultimately contained, ongoing epidemiologically linked larger outbreaks are occurring in other states (*6*,*12*,*13*). Because acute care hospitals play an increasing role in outbreak-control programs, close coordination between public health and acute care hospitals, as well as optimization of informatic tools to improve the identification of at-risk population, can contribute substantially to control of communitywide outbreaks.

Appendix Twinrix vaccinations administered during community outbreak of hepatitis A, San Diego County, California, USA.

## References

[1] Bennett JE, Dolin R, Blaser MJ. Mandell, Douglas, and Bennett’s principles and practice of infectious diseases. 8th ed. Amsterdam: Saunders; 2015.

[2] Wasley A, Samandari T, Bell BP. Incidence of hepatitis A in the United States in the era of vaccination. JAMA. 2005;294:194–201. 10.1001/jama.294.2.194 16014593

[3] Murphy TV, Denniston MM, Hill HA, McDonald M, Klevens MR, Elam-Evans LD, et al. Progress toward eliminating hepatitis A disease in the United States. MMWR Suppl. 2016;65:29–41. 10.15585/mmwr.su6501a6 26916458

[4] André FE, D’Hondt E, Delem A, Safary A. Clinical assessment of the safety and efficacy of an inactivated hepatitis A vaccine: rationale and summary of findings. Vaccine. 1992;10(Suppl 1):S160–8. 10.1016/0264-410X(92)90576-6 1335652

[5] California Department of Public Health. Hepatitis A [cited 2019 Apr 16]. https://www.cdph.ca.gov/Programs/CID/DCDC/pages/immunization/hepatitis-a.aspx

[6] Centers for Disease Control and Prevention. Widespread outbreaks of hepatitis A across the United States [cited 2019 Nov 11]. https://www.cdc.gov/hepatitis/outbreaks/2017March-HepatitisA.htm

[7] County of San Diego. Hepatitis A outbreak after action report [cited 2019 Apr 16]. https://www.sandiegocounty.gov/content/dam/sdc/cosd/SanDiegoHepatitisAOutbreak-2017-18-AfterActionReport.pdf

[8] Castillo EM, Chan TC, Tolia VM, Trumm NA, Powell RA, Brennan JJ, et al. Effect of a computerized alert on emergency department hepatitis A vaccination in homeless patients during a large regional outbreak. J Emerg Med. 2018;55:764–8. 10.1016/j.jemermed.2018.09.004 30316620

[9] Mbithi JN, Springthorpe VS, Sattar SA. Chemical disinfection of hepatitis A virus on environmental surfaces. Appl Environ Microbiol. 1990;56:3601–4. 10.1128/AEM.56.11.3601-3604.1990 2176450 PMC185032

[10] Centers for Disease Control and Prevention. National Notifiable Diseases Surveillance System (NNDSS). Hepatitis A, acute 2012 case definition [cited 2019 Nov 1]. https://wwwn.cdc.gov/nndss/conditions/hepatitis-a-acute/case-definition/2012/

[11] Kushel M. Hepatitis A outbreak in California—addressing the root cause. N Engl J Med. 2018;378:211–3. 10.1056/NEJMp1714134 29211622

[12] Foster M, Ramachandran S, Myatt K, Donovan D, Bohm S, Fiedler J, et al. Hepatitis A virus outbreaks associated with drug use and homelessness—California, Kentucky, Michigan, and Utah, 2017. MMWR Morb Mortal Wkly Rep. 2018;67:1208–10. 10.15585/mmwr.mm6743a3 30383739 PMC6319801

[13] Foster MA, Hofmeister MG, Kupronis BA, Lin Y, Xia GL, Yin S, et al. Increase in hepatitis A virus infections—United States, 2013–2018. MMWR Morb Mortal Wkly Rep. 2019;68:413–5. 10.15585/mmwr.mm6818a2 31071072 PMC6542191

[14] Doshani M, Weng M, Moore KL, Romero JR, Nelson NP. Recommendations of the Advisory Committee on Immunization Practices for use of hepatitis A vaccine for persons experiencing homelessness. MMWR Morb Mortal Wkly Rep. 2019;68:153–6. 10.15585/mmwr.mm6806a6 30763295 PMC6375653

[15] Peak CM, Stous SS, Healy JM, Hofmeister MG, Lin Y, Ramachandran S, et al. Homelessness and Hepatitis A - San Diego County, 2016-2018. Clin Infect Dis. 2019;•••:ciz788. 10.1093/cid/ciz788 31412358 PMC10956402

[16] Duncan L. A community clinic’s response to a hepatitis A outbreak. Am J Infect Control. 2018;46:1057–9. 10.1016/j.ajic.2018.02.007 29555142

[17] James TL, Aschkenasy M, Eliseo LJ, Olshaker J, Mehta SD. Response to hepatitis A epidemic: emergency department collaboration with public health commission. J Emerg Med. 2009;36:412–6. 10.1016/j.jemermed.2007.10.001 18359602

[18] Rimple D, Weiss SJ, Brett M, Ernst AA. An emergency department-based vaccination program: overcoming the barriers for adults at high risk for vaccine-preventable diseases. Acad Emerg Med. 2006;13:922–30. 16902048 10.1197/j.aem.2006.04.022

[19] Pahud B, Clark S, Herigon JC, Sherman A, Lynch DA, Hoffman A, et al. A pilot program to improve vaccination status for hospitalized children. Hosp Pediatr. 2015;5:35–41. 10.1542/hpeds.2014-0027 25554757

[20] Robke JT, Woods M. A decade of experience with an inpatient pneumococcal vaccination program. Am J Health Syst Pharm. 2010;67:148–52. 10.2146/ajhp080638 20065270

[21] Middleton DB, Fox DE, Nowalk MP, Skledar SJ, Sokos DR, Zimmerman RK, et al. Overcoming barriers to establishing an inpatient vaccination program for pneumococcus using standing orders. Infect Control Hosp Epidemiol. 2005;26:874–81. 10.1086/502511 16320983

[22] Lindegren ML, Atkinson WL, Farizo KM, Stehr-Green PA. Measles vaccination in pediatric emergency departments during a measles outbreak. JAMA. 1993;270:2185–9. 10.1001/jama.1993.03510180055033 8411600

[23] Dexter PR, Perkins SM, Maharry KS, Jones K, McDonald CJ. Inpatient computer-based standing orders vs physician reminders to increase influenza and pneumococcal vaccination rates: a randomized trial. JAMA. 2004;292:2366–71. 10.1001/jama.292.19.2366 15547164

[24] Dexter PR, Perkins S, Overhage JM, Maharry K, Kohler RB, McDonald CJ. A computerized reminder system to increase the use of preventive care for hospitalized patients. N Engl J Med. 2001;345:965–70. 10.1056/NEJMsa010181 11575289

[25] Centers for Disease Control and Prevention. Hepatitis A [cited 2019 Jul 26]. https://www.cdc.gov/hepatitis/HAV/HAVfaq.htm#general

[26] Daniels D, Grytdal S, Wasley A; Centers for Disease Control and Prevention (CDC). Surveillance for acute viral hepatitis - United States, 2007. MMWR Surveill Summ. 2009;58:1–27. 19478727

[27] Wasley A, Miller JT, Finelli L; Centers for Disease Control and Prevention (CDC). Surveillance for acute viral hepatitis—United States, 2005. MMWR Surveill Summ. 2007;56:1–24. 17363893

[28] Wasley A, Grytdal S, Gallagher K; Centers for Disease Control and Prevention (CDC). Surveillance for acute viral hepatitis—United States, 2006. MMWR Surveill Summ. 2008;57:1–24. 18354374

[29] Collier MG, Tong X, Xu F. Hepatitis A hospitalizations in the United States, 2002-2011. Hepatology. 2015;61:481–5. 10.1002/hep.27537 25266085

