# Supplementary material for: Public Health Role of Academic Medical Center in Community Outbreak of Hepatitis A, San Diego County, California, USA, 2016–2018
Source: Emerg Infect Dis. 2020 Jul;26(7):1374–81. doi: 10.3201/eid2607.191352 (PMC7323565; doi:10.3201/eid2607.191352)
Supplement: Appendix — Twinrix vaccinations administered during community outbreak of hepatitis A, San Diego County, California, USA. [file 19-1352-Techapp-s1.pdf]

Article DOI: https://doi.org/10.3201/eid2607.191352

Public Health Role of Academic Medical Center in Community Outbreak of Hepatitis A, San Diego County, California, USA, 2016–2018

Appendix

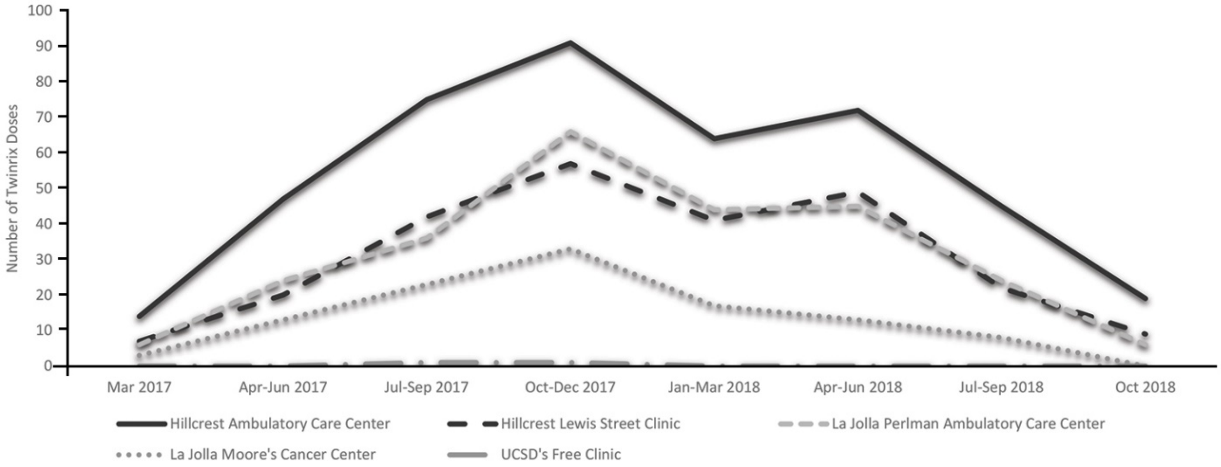

Appendix Figure. Twinrix (GlaxoSmithKline, https://www.gsksource.com) doses administered at ambulatory care clinic at University of California San Diego Health.
